# Perceived stress among university students in south-east Serbia during the COVID-19 outbreak

**DOI:** 10.1186/s12991-021-00346-2

**Published:** 2021-04-07

**Authors:** Jelena Kostić, Olivera Žikić, Vladimir Đorđević, Žilijeta Krivokapić

**Affiliations:** 1 Department of Psychiatry, Faculty of Medicine, University of Niš, Niš, Serbia; 2 Centre for Mental Health Protection, Clinical Center Niš, Niš, Serbia; 3 College of Health Studies, Ćuprija, Serbia

**Keywords:** Students, Perceived stress, COVID-19

## Abstract

**Background:**

The COVID-19 pandemic has brought into focus the mental health of the student population. The study aimed to analyze the psychological response to the COVID-19 outbreak in terms of perceived stress and its related factors among university students in south-east Serbia. The study was conducted during the increased incidence of COVID-19 in Serbia.

**Method:**

A total of 434 students from the public university in south-east Serbia enrolled in the study and completed the measures of socio-demographic data, the perceived stress scale (PSS-10), the Coping Strategy Indicator (CSI) and the General Health Questionnaire (GHQ-28). The data were analyzed through quantitative and qualitative methods.

**Results:**

Study findings suggest that the mean perceived stress score was placed to 20.43 (± 7.67). Our model showed that female gender, higher scores on anxiety/insomnia and depression subscale as well as the coping strategy avoidance predicted higher perceived stress, while higher scores on social dysfunction were related to the reduced perceived stress scores.

**Conclusion:**

Notwithstanding the study limitation, findings provided authentic data of stress reactions of the students in south-east Serbia during the COVID-19 outbreak. The findings confirm the need to examine students' experiences in emergencies and crises, as well as to make a plan for online stress management programs that would help alleviate stress during a global pandemic.

## Introduction

On March 11th 2020, the World Health Organization (WHO) declared COVID-19 a pandemic [1]. On March 15th, a state of emergency was introduced in Serbia to prevent the spread of the infection and lasted until May 6th 2020. Since most people do not know about emerging infections in the first stages, they are more affected by social and psychological problems, generally causing widespread fear and irrational reactions to it [2]. The student population in Serbia faced sudden disruptions in their academic study, universities closing down, distance learning, reduced social interactions as well as strict public health measurements (including the stay-at-home orders, quarantine, and isolation to reduce social contacts). Some papers pointed out that an infectious disease outbreak can represent a significant psychological stressor and lead to unfavorable effects on learning and the overall psychological students’ health [3].

Stress can be defined as “a particular relationship between the person and the environment that is appraised by the person as taxing or exceeding his/her resources and endangering his/her well-being” [4]. Stress is associated with negative physical health outcomes and the exacerbation of mental health symptoms and psychological distress [5, 6]. Psychological distress is largely defined as a state of emotional suffering characterized by symptoms of depression and anxiety [7]. These symptoms often coexist and co-occur with common somatic complaints [8]. It has been established that coping is a key variable in the process of reducing, minimizing, or tolerating stress [9] as well as preventing psychological distress [10]. Coping strategies refer to behavioural and cognitive efforts that help reduce the pressure of a stressful situation and are used when its demands exceed individual resources [4]. The appraisal literature describes the response or coping process in terms of problem-focused or emotion-focused coping [4], also referred to as *active* and *avoidance* coping styles [11]. Active coping is characterized by strategies such as problem-focused coping and is generally associated with more adaptive adjustment, whereas passive coping (such as negative self-targeting and avoidance) is represented as a maladaptive strategy when facing stressful situations [12]. Based on the previous experiences with infectious disease outbreaks, a study among 381 undergraduate students suggested that the number of stressors and the use of avoidant coping strategies during the 2003 Beijing SARS epidemic predicted psychological symptoms, whereas active coping strategies predicted life satisfaction [13].

Current research of the psychological impact of the COVID-19 pandemic on students is related to Asian and Western samples [14–20]. The main goal of this study was to investigate the psychological response of university students in south-east Serbia to the COVID-19 outbreak in terms of perceived stress and its related factors. The perception of stress, i.e. the degree to which one perceives the situation as being stressful [21], accounts for the varying responses to potentially stressful events. We explored the association of gender, psychosocial stressors common to COVID-19, coping strategies for dealing with stressful situations and psychological distress levels with perceived stress during the coronavirus outbreak.

## Method

The study was approved by the Board of Ethics at the Clinical Centre Nis, University of Nis (i.e. the regulatory Authority providing the guidelines for research and clinical practice). Students at the University of Nis were informed about the purpose of this anonymous survey and invited to participate in the study via social media. Data collection for the study was accomplished by distributing the survey instruments online through Google forms together with an appended consent form. The study was conducted during the increasing incidence of COVID-19 cases from 18th May to 1st June 2020.

The participants filled in the demographic information on age, gender, and living conditions. They were given a few closed questions (yes/no) about psychosocial stressors common to COVID-19–infected by SARS-CoV-2, feelings of concern and fear of contracting COVID-19, concern for the health of family members, taking part in volunteer activities to help disabled persons during the coronavirus outbreak. Three main outcome measures were used:

### Perceived stress scale (PSS-10) [21]

This scale evaluates the respondents’ perceptions of stress levels they experience in specific situations. The respondents answered ten questions on how unpredictable, uncontrollable, and overloaded they found their lives to be during the previous month, which was suitable for the current situation with the Corona 19 outbreak. In each case, the respondents were asked how often they had felt a certain sensation in the previous month. A 5-point Likert scale (ranging from 0 = ”never” to 4 = ”very often”) was used to grade the levels of perceived stress. The global PSS score ranges from 0 to 40 with higher scores indicating higher levels of perceived stress. A score ranging from 0–13 would be considered low stress; a score between 14 and 26 represents moderate stress. A score ranging from 27–40 represents a high level of perceived stress. There is a norm table for the PSS-10 item inventory**.**

### General health questionnaire (GHQ-28) [22]

Psychological distress levels were measured through the GHQ-28. This 28-item self-administered questionnaire is grouped into four subscales: somatic symptoms, anxiety/insomnia, depression, and social dysfunction. The scoring system in this study was the same as the original scoring system, the 4-point Likert scoring method ranging from 0 = “better than usual” to 3 = “much worse than usual”. The minimum score for the GHQ-28 version is 0, and the maximum is 84. Higher GHQ-28 scores indicate a greater probability of psychological distress. Total scores of 23 or below should be classified as non-psychiatric, while scores ≥ 24 indicate the presence of psychological distress or “caseness” but this score is not an absolute cut-off.

### The coping strategy indicator (CSI) [23]

Is a 33-item, 3-point self-report rating scale designed to assess three separate, large, heterogeneous fundamental strategies: problem-solving (PS), seeking social support (SS), and avoidance (A). Problem-solving involves an instrumental, problem-oriented approach to managing stressors actively. Seeking social support relates to the basic human need for human contact in times of forcible restraint and is manifested by actively seeking comfort, help, and advice from others. Avoidance involves escape responses, such as physical and/ or psychological withdrawal, for example, through distraction or fantasy. The items of the CSI are scaled on a 3-point Likert scale (1 = “not at all”, 3 = “a lot”). These responses indicate whether participants cope by problem-solving, seeking social support or avoiding the event. Higher scores on each subscale suggest a higher probability to make use of the associated coping strategy. There are CSI norms established through the initial CSI validation study.

### Data analysis

SPSS software version 15.0 was used for statistical data processing. The frequencies of the qualitative features were measured by the |^2^ test. Upon determining the normal distribution and testing normality using the Kolmogorov–Smirnov test, the values of continuous variables were compared among different modalities using the Student’s *t* test (for independent samples) in the case of normal distribution, or the Mann–Whitney *U* test in case the distribution deviated from normal. The Spearman's rank correlation coefficient was used to determine the interconnection of continuous parameters. The factors important for the prediction of the PSS score were determined by the univariate and Stepwise multivariate linear regression analysis.

## Results

434 students completed the questionnaire. SARS-CoV-2 infection was confirmed in six respondents (1.38%) and these were excluded from further statistical data processing.

The sample included a statistically relevant difference in the number of female respondents (335, i.e. 78.27%) in comparison to the male (*p* < 0.001). The average respondents ‘age was 23.81 ± 5.25 (range 19 25). The statistically relevant majority, 324 (75.50%) lives with their parents.

Table 1. shows the structure of the respondents by gender, age and place of residence.

**Table 1:** Respondents' structure by gender, age, and the place of residence

Parameter	*n*	%	*p*
Gender			
Male	93	21.73%	
Female	335	78.27%	***
Age	23.81 ± 5.25 (23.00) [19–25]		
Residence			
Living with the parents	324	75.50%	***
Other	104	24.30%	

Continuous variables are represented as *X* ± SD (Me) [min–max], and the category variables in absolute numbers and percentage ***p < 0.001 (Pearson’s *χ*^2^test)

Table 2 shows that significantly fewer respondents 71 (16.59%) reported feelings of concern and fear of contracting COVID-19, significantly more respondents 263 (61.45%) had concerns for the health of family members, whereas significantly fewer respondents, 49 (11.45%), participated in any volunteer activities (*p* < 0.001).

**Table 2:** Respondent’s answers on psychosocial stressors common to COVID-19

Question and the offered answers	*n*	%	*p*
Feelings of concern and fear of contracting COVID-19			
Yes	71	16.59%	
No	357	83.41%	***
Concern for the health of family members			
Yes	263	61.45%	***
No	165	38.55%	
Participation in volunteer activities			
Yes	49	11.45%	
No	379	88.55%	***

The frequency of category variables is represented in absolute numbers and percentage; *** -p < 0.001 (Pearson’s χ^2^ test)

Table 3. shows the values of the descriptive parameters for the PSS-10, CSI and GHQ-28 scores among the non-infected respondents (*n* = 428).

**Table 3:** Descriptive statistic for the PSS-10, CSI, and GHQ-28 scores (n = 428)

Scale		
PSS–10	20.37 ± 7.62	(20.00)
CSI		
Problem-solving	25.76 ± 4.61	(26.00)
Seeking social support	22.15 ± 5.01	(22.00)
Avoidance	23.78 ± 4.20	(24.00)
GHQ-28		
Somatic symptoms	6.56 ± 4.75	(6.00)
Anxiety	8.03 ± 5.91	(7.00)
Social dysfunction	7.99 ± 3.72	(7.00)
Depression	3.69 ± 5.04	(1.00)
GHQ-28 total	26.28 ± 14.52	(23.00)
GHQ-28 total (≥ 24)	209	(48.83%)

Continuous variables are represented as *X* ± SD (Me)

Qualitative parameters GHQ-28 total (≥ 24) are shown in frequency and %

^***^p < 0.001 (Pearson’s *χ*^2^test)

Based on the average values on the PSS-10 of 20.37 ± 7.62, it is evident that the average level of perceived stress is moderate because it is between 14 and 26, which defines it as such based on this scale [21] (Table 3).

Based on the average values of the CSI scores, the value of 25.76 ± 4.61 (26.00) on problem-solving is within the expected average of 26, seeking social support, 22.15 ± 5.01 (22.00), is slightly lower than the expected average of 23.00, whereas the score on avoidance of 23.78 ± 4.20 (24.00) is significantly above the expected average of 19 (Table 3).

The findings on the GHQ-28 identified that 48.83% of students scored higher or equal to 24 (Table 3). On the GHQ-28, the highest average values were on the anxiety/insomnia subscale, with the average value of 8.03 ± 5.91 (7.00), closely followed by the social dysfunction subscale with 7.99 ± 3.72 (7.00). The score on somatic complaints was significantly lower, 6.56 ± 4.75 (6.00), while the least pronounced was the depression subscale, with 3.69 ± 5.04 (1.00) (Table 3).

The Student's *t* test (for independent samples) revealed a significantly higher PPS in female respondents (*p* < 0.01), respondents who expressed concern for the health of family members (*p* < 0,01) and in students who did not participate in any volunteer activities (*p* < 0.001) (Table 4).

**Table 4:** Descriptive parametric statistics for the PSS-10 in relation to the place of residence, feelings of concern and fear of contracting COVID-19, high-risk family members and volunteering

PSS		
Gender		
Male (*n* = 93)	18.29 ± 7.19	(19.00)
Female (*n* = 335)	20.95 ± 7.64	(21.00)^**^
Living with parents		
Yes (*n* = 324)	20.30 ± 7.71	(20.00)
No (*n* = 104)	20.59 ± 7.35	(21.00)
Feelings of concern and fear of contracting COVID-19		
Yes (*n* = 71)	20.59 ± 9.21	(23.00)
No (*n* = 357)	20.33 ± 7.27	(20.00)
Concern for the health of family members		
Yes (*n* = 263)	21.32 ± 7.46	(21.00)^**^
No (*n* = 165)	18.86 ± 7.64	(19.00)
Volunteering during the COVID-19 outbreak		
Yes (*n* = 49)	16.80 ± 7.66	(17.00)
No (*n* = 379)	20.83 ± 7.50	(21.00)^***^

Continuous variables are represented as *X* ± SD (Me)

^**^p < 0.01, ***p < 0.001 (Student’s t test for independent samples)

The Spearman's rank correlation coefficient showed a statistically relevant negative and low correlation of the PSS-10 values with problem-solving (*ρ* =–0.21, *p* < 0.001) and a positive and high statistically relevant correlation of PSS with avoidance (*ρ* = 0.50, *p* < 0.001). There was no relevant correlation between the PSS values and seeking social support (*ρ* =–0.001, *p* > 0.05).

The Spearman's rank correlation coefficient showed a statistically relevant positive and high correlation of the PSS-10 values with somatic complaints (*ρ* = 0.643, *p* < 0.001) and depression (*ρ* = 0.645, *p* < 0.001), and an even higher positive correlation with anxiety/insomnia (*ρ* = 0.763, *p* < 0.001).

The results of the univariate linear regression analysis showed that the higher PSS-10 values were significantly influenced by the overall GHQ-28 scores higher or equal to 24, the overall GHQ-28 questionnaire scores alone, and the scores on its three subscales (somatic symptoms, anxiety/insomnia, and depression). The avoidance subscale on the CSI scale, concern for the health of family members, and the female gender proved to influence the results further (Table 5).

**Table 5.** Assessment of the impact of factors of interest on the PSS-10 score, the results of the univariate linear regression analysis

Factor	*p*	*B*	95% CI for *B*	
			Lower	Lower
Living with parents	0.7411	− 0.284	− 1.973	1.405
Female	0.0028	2.659	0.921	4.397
Feelings of concern and fear of contracting COVID-19	0.7902	0.264	− 1.684	2.211
Concern for the health of family members	0.0011	2.459	0.989	3.929
Volunteering—helping disabled persons	0.0004	− 4.038	− 6.280	− 1.795
Problem-solving	0.0000	− 0.393	− 0.546	− 0.240
Seeking social support	0.9800	0.002	− 0.143	0.147
Avoidance	0.0000	0.880	0.729	1.031
Somatic symptoms	0.0000	0.983	0.862	1.104
Anxiety/insomnia	0.0000	0.959	0.877	1.040
Social dysfunction	0.0325	− 0.212	− 0.406	− 0.018
Depression	0.0000	0.871	0.754	0.989
GHQ-28 Total Score	0.0000	0.355	0.319	0.392
GHQ-28 Total Score ≥ 24	0.0000	8.709	7.521	9.898

B–Regression coefficient, CI–trust interval

The overall GHQ-28 questionnaire score higher or equal to 24 increased the PSS-10 score for 8.71 (7.521–9.898, *p* < 0.001) in comparison to the respondents with an overall GHQ-28 up to 23. This has the highest influence on the PPS-10 (Table 5).

Female respondents had a 2.659 (0.921–4.397, *p* < 0.01) higher PSS scores in comparison to the male ones, whereas the ones with a concern for the health of family members had a 2.459 (0.989–3.929, *p* < 0.01) higher PPS scores in comparison to the respondents who expressed no concern for the health of family members (Table 5).

A unit increase on the somatic complains subscale score led to an increase in the PSS score by 0.983 (0.862–1.104, *p* < 0.001); a unit increase on the anxiety/insomnia subscale score led to an increase in the PSS score by 0.959 (0.877–1.040, *p* < 0.001) and on depression by 0.871 (0.754–0.989, *p* < 0.001). A unit increase on the avoidance subscale led to an increase in the PSS score by 0.880 (0.729–1.031, *p* < 0.001) (Table 5).

A unit increase of the overall GHQ-28 score led to an increase in the PSS score by 0.355 (0.319–0.392, *p* < 0.001) (Table 5).

Volunteering—helping the disabled persons during the COVID-19 pandemic had a statistically relevant influence on lowering the PSS scores by 4.038 (− 1.795 to − 6.280, *p* < 0.001) compared with the non-volunteers. Apart from volunteering, a statistically relevant influence on lowering the PSS scores proved to be the sub-score problem-solving on the CSI questionnaire (its unit increase lowers the PSS score by 0.393 (− 0.240 to − 0.546, *p* < 0.001)) and Social dysfunction, the sub-score on the GHQ-28 scale, whose unit increase lowered the PSS-10 score by 0.212 (− 0.018 to − 0.406, *p* < 0.05) (Table 5).

The initial model of multivariate linear regression analysis was formed based on the variables that were shown in the univariate analysis as factors with a significant influence on the PSS scores. By applying the stepwise regression in step 5, an optimal model of the combined influence of the variables on the PSS-10 score was obtained. This consisted of anxiety/insomnia, depression, gender, avoidance, and social dysfunction (Table 6). The multiple-correlation coefficient *R* is 0.783, and the multiple-determination coefficient is 0.613, which means that in 61.3% of the tested sample, the PSS-10 score variance was determined by the variance of the set of predictor variables found in the final model. The female gender showed to be the most significant factor influencing the increase in the PSS score, followed by anxiety/insomnia, depression and avoidance, whereas social dysfunction showed to decrease the score we examined (Table 6).

**Table 6.** Assessment of the impact of factors of interest on the PSS-10 score, the results of the multivariate linear regression analysis

Factor	*p*	*B*	95% CI for *B*	
			Lower	Upper
Anxiety	0.0000	0.739	0.634	0.843
Depression	0.0000	0.293	0.178	0.408
Social dysfunction	0.0001	− 0.241	− 0.363	− 0.118
Female	0.0007	1.913	0.808	3.017
Avoidance	0.0079	0.174	0.046	0.302
(Constant)	0.0000	9.650	6.585	12.715

B–Regression coefficient, CI–trust interval, *R*^2^ = 0.613

## Discussion

This study aims to evaluate the perceived stress and its related factors due to the COVID-19 outbreak among university students in south-east Serbia. The mean perceived stress score of 20.37 (± 7.67) suggests that our participants had relatively high levels of perceived stress compared with established norms for a general population sample aged 18–29 (*M* = 14.2 (± 6.2)) [21]. Furthermore, the PSS results were higher than the mean norms for PSS of 14.98 (± 6.32), obtained in an earlier pre-COVID-19 survey among Serbian students aged 21.82 [24].

Study results are in line with recent studies which showed moderate levels of perceived stress among the student population [14, 20, 25] during the COVID-19 outbreak. For example, Sheroun et al. [14] have found that the mean perceived stress score of the college student Nurses in Puna, aged 21–25, was 21.88 (± 4.30). Collecting the data about the perceived stress during the COVID-19, an internet survey conducted among 2449 residents in 20 provinces of China, reports that students had the strongest perceived stress of 23.87 (± 6.18) compared with other occupations and that in 48.66%, stress represented a health risk [25]. Son et al. [20] reported the mean PSS score of 18.8 (± 4.9) among students, average age approximately 20.7, indicating moderate perceived stress during the outbreak of the coronavirus disease.

Our findings showed that female students were significantly more stressed during the COVID-19 outbreak than the male and that being female significantly predicted higher perceived stress in our sample. Earlier research [3] as well as the recent studies carried out on the student population during the COVID-19 [15, 16, 26] have shown significant gender differences in the psychological response to the epidemic. Mirowsky and Ros [7] found in their study that gender influences the appraisal process of stressful events in ways that are consistent with the different socialization patterns of males and females.

In the present study, expressing concern for the health of family members during the COVID-19 outbreak predicted higher levels of perceived stress. Recent studies found that concerns relating to the health of family members have been highly prevalent among the student population during the pandemic [20, 27]. Most of the students in our sample live with their parents. Since it is assumed that older people are more likely to have a worse prognosis after a SARS-CoV-2 infection, students may have been more concerned about their parents and other older family members than about themselves. Ding et al. [27] pointed out that students believe their parents have a higher risk of being infected by COVID-19 and that their own risk is lower because the parents have to go to work or take care of the family. Furthermore, students cannot control their parents’ behavior [27].

Many positive benefits have been attributed to a volunteer role. It is stated that volunteers experience a “helper’s high”—a prolonged feeling of calm, reduced stress, and greater self-worth after helping others [28]. Some negative consequences to volunteering have also been noted, such as stress and burnout [29]. Our findings indicate accordingly that students involved in volunteering activities helping the disabled persons during the COVID-19 outbreak, experience lower perceived stress than the non-volunteers. It is still unclear whether volunteering reduces the stress level or students who perceived lower stress are simply more likely to volunteer during an actual pandemic.

Among the three basic models of CSI coping, our study findings show that problem-solving was most used in response to the COVID-19 emergency, followed by avoidance and seeking social support. On the other hand, our findings suggest that the mean scores obtained on the avoidance subscale are significantly above the expected normative average for CSI. Previous studies during the SARS epidemic (2002–2004) reported that college students used less active (problem-focused) coping strategies and more avoidant coping strategies in response to SARS-related stressors (which were rated by participants as less controllable) in comparison to daily stressors during the outbreak [30]. The correlation analyses and our model showed that avoidance coping not only has a significant positive correlation but also serves as a good predictor of perceived stress. These results are in line with previous studies which reported the negative impact of the avoidance strategy on university adjustment, especially the mental health of students [31, 32] and the current literature on the relationship between coping and response to epidemics [33, 34]. Despite the efficacy and potential benefits derived from employing avoidance coping under specific situations (such as in situations that require immediate and short-term responses to threat as well as those that are uncontrollable [4]), this coping mechanism is grouped among dysfunctional reactions to stressful situations as it deals with the likelihood with which individuals adopt strategies based on avoidance when they face problematic situations. Regarding the problem-solving strategy, our results show a negative and significant correlation between perceived stress and problem-solving. These results are consistent with other past and current studies on infectious disease outbreaks indicating that problem-solving is an active coping strategy with a psychological impact on stress reduction [13, 33–35]. Some research during the current coronavirus pandemic among the general Italian population has shown that seeking social support was positively related to perceived stress [33] and that higher social support predicted higher levels of stress [34]. In this study, a negative correlation was found between seeking social support and perceived stress, but not at the level of statistical significance.

Using the cut-off score of GHQ-28, it was found that 48.83% of students reported psychological distress. This seems to suggest that the students are psychologically healthy in general but a considerable percentage of them have been identified to have the potential to develop psychological problems. Psychological distress analysis using GHQ-28 during the COVID-19 outbreak showed the highest average scores among students on the anxiety/insomnia subscale, followed by social dysfunction and somatization, whereas the lowest average values were recorded on the depression subscale. According to Leung et al., large outbreaks of novel or serious infectious diseases are associated with levels of anxiety that may be far greater than the risk of becoming infected or of mortality from infection [35]. Recent studies, which evaluated the mental health of university students during COVID-19 in Spain [16] and China [15, 17] reported anxiety, stress, and depression.

Obtained positive correlation among PSS-10 scores and somatic complaints, depression, and anxiety are expected. Some authors argued that the PSS is “yet another measure of psychopathology or distress,” [36] suggesting the problem of circularity and overlap between the measures of perceived stress and emotional distress. Indeed, it is difficult to make a distinction between perceived stress and emotional distress, because they share a common thread of unpleasant emotions. But there is a conceptual difference between perceived stress as measured by the PSS and emotional distress. Perceived stress primarily refers to the cognitive evaluation of stress, i.e., “the degree to which situations in one’s life is appraised as stressful” [21]. On the other hand, emotional distress refers to the mental health outcome variable, i.e., negative emotional consequences that may result from numerous factors other than perceived stress. Therefore, perceived stress does not necessarily result in negative outcomes (e.g., in high resilient people), while emotional distress is inherently negative.

Regression analysis indicates that an increase in distress indicators, marked by a GHQ-28 total score higher or equal to 24, together with its three factors including anxiety/insomnia, depression, and somatic symptoms lead to a significant increase in the stress level in our sample. The multivariable model showed that anxiety/insomnia and depression are strongly associated with perceived stress, indicating a common mechanism between mental health disorders and stress confrontation. Contrary to the findings in the literature, which indicate a positive association between the social dysfunction subscale on the GHQ-28 and perceived stress among the student population [37], our research showed that higher scores on the social dysfunction subscale predicted lower perceived stress. The social dysfunction subscale is comprised of items that indicate engaging in everyday activities like “been satisfied with the way you’ve carried out your task”, “been taking longer over the things you do”, where higher values recorded on this subscale suggest compromised functional abilities of the respondents. A possible explanation of this result may be related to the dramatic situation associated with the COVID-19 pandemic and up to now unseen global reaction to some disease. Namely, soon after the declaration of the pandemic, the University of Nis closed all facilities, suspended classes, exam deadlines, and almost all academic obligations. The student population is under constant pressure to meet the set deadlines and coordinate studies with other obligations, they are constantly chasing grades, fighting busy schedules, and facing a lack of free time. It is, therefore, possible that in the circumstances when “the whole world stopped because of the coronavirus” students were less satisfied with the way they performed their tasks, but at the same time had an external justification for the reduced functional performance, and were relieved and under less stress. This result has to be observed with caution as further research is needed to explore it, perhaps with control measures such as years of studying, previous success in studies, perceived self-efficacy, etc.

## Limitation

There are several limitations to the study. The study sample was small and the survey included only university students in the southeastern region of Serbia, thus being hardly representative of all Serbia. Recruiting more students from different regions of the country could improve the study results. Also, an online assessment may entail data of a lower quality than that obtained by face-to-face interviews. Finally, the cross-sectional design of the study has a limitation in determining a causal relationship between factors of interest and perceived stress and evaluating the level of stress during the actual pandemic and pre-COVID-19 period.

## Conclusion

Study findings suggest that PSS results showed moderate levels of stress among our participants during the COVID-19 outbreak. Our model there showed that female respondents had higher scores on anxiety/insomnia and depression subscales on GHQ-28 and that avoidance coping was strongly associated with perceived stress, while higher scores on the social dysfunction subscale reduced stress. The nature of the association between social dysfunction and perceived stress could be the subject of further research. The findings of the study confirm the need to examine the experience of students in states of emergencies and crises as well as make a plan for online stress management programs that would help alleviate stress during a global pandemic. Besides, further studies are needed to determine the effects of the pandemic on the mental health of students in the later stages of a health crisis.

## Data Availability

All data generated or analyzed during this study are included in this published article.

## Abbreviations

PSS: Perceived stress scale-10
CSI: Coping Strategy Indicator
GHQ: General Health Questionnaire
